# IL-27 alters inflammatory cytokine expression and limits protective immunity against *Mycobacterium tuberculosis* in a neonatal BCG vaccination model

**DOI:** 10.3389/fimmu.2024.1217098

**Published:** 2024-02-08

**Authors:** Shelby D. Bradford, Kenneth J. Ryan, Ashley M. Divens, Jessica M. Povroznik, Sunilkanth Bonigala, Cory M. Robinson

**Affiliations:** ^1^ Department of Microbiology, Immunology, & Cell Biology, West Virginia University School of Medicine, Morgantown, WV, United States; ^2^ Vaccine Development Center, West Virginia University Health Sciences Center, Morgantown, WV, United States; ^3^ Department of Statistics, West Virginia University, Morgantown, WV, United States

**Keywords:** interleukin-27, neonate, BCG, tuberculosis, vaccine, vaccination, cytokine

## Abstract

**Background:**

Efforts to control tuberculosis (TB), caused by the pathogen *Mycobacterium tuberculosis* (Mtb), have been hampered by the immense variability in protection from BCG vaccination. While BCG protects young children from some forms of TB disease, long-term protection against pulmonary disease is more limited, suggesting a poor memory response. New vaccines or vaccination strategies are required to have a realistic chance of eliminating TB disease. In TB endemic areas, routine immunization occurs during the neonatal period and as such, we hypothesized that inadequate protective immunity elicited by BCG vaccination could be the result of the unique early-life immune landscape. Interleukin (IL)-27 is a heterodimeric cytokine with immune suppressive activity that is elevated in the neonatal period.

**Objective:**

We investigated the impact of IL-27 on regulation of immune responses during neonatal BCG vaccination and protection against Mtb.

**Methods:**

Here, we used a novel model of neonatal vaccination and adult aerosol challenge that models the human timeline of vaccine delivery and disease transmission.

**Results:**

Overall, we observed improved control of Mtb in mice unresponsive to IL-27 (IL-27Rα^-/-^) that was consistent with altered expression patterns of IFN-γ and IL-17 in the lungs. The balance of these cytokines with TNF-α expression may be key to effective bacterial clearance.

**Conclusions:**

Our findings suggest the importance of evaluating new vaccines and approaches to combat TB in the neonatal population most likely to receive them as part of global vaccination campaigns. They further indicate that temporal strategies to antagonize IL-27 during early life vaccination may improve protection.

## Introduction

Tuberculosis, caused by *Mycobacterium tuberculosis* (Mtb), remains one of the leading causes of death from an infectious agent with 1.6 million deaths reported worldwide in 2021 (1). Bacille Calmette Guérin (BCG), the only preventative measure to combat tuberculosis is one of the most utilized vaccines in the world, with approximately 4 billion doses estimated to have been given throughout history (2). BCG successfully protects infants and young children from pulmonary and disseminated disease. Unfortunately, long-term protection afforded by BCG is highly variable at preventing pulmonary disease in adolescence and adulthood, and this is indicated as a contributing factor to continued transmission in TB endemic regions (3, 4). Several hypotheses exist for why BCG does not provide adequate long-term protection. Among these, our interest is in the age of vaccination and subsequent immune landscape of the recipient, as BCG is one of only two vaccines routinely given in the neonatal period (5). BCG is most often administered at the time of birth in TB endemic areas and this is a time at which the neonatal immune system is described as “immune tolerant”. This immune profile is characterized by a reduced expression of stimulatory receptors, a bias toward Th2 responses, and a skew to anti-inflammatory cytokines over proinflammatory cytokines (6).

Interleukin (IL)-27 is a heterodimeric cytokine of the IL-12 family with immune suppressive activity and has been shown to be elevated in the neonatal period (7–9). Previous studies that investigated the involvement of IL-27 in protection against TB demonstrated an inhibitory role toward pathogen control, with mice unresponsive to IL-27 exhibiting improved Mtb clearance (10–13). However, it is important to note that improved pathogen control was accompanied by heightened inflammation and tissue damage in these studies and those addressing other chronic infections (11, 14). This further points to a primary suppressive role of IL-27 in response to infection or vaccination with live agents. IL-27 has also been shown to negatively regulate IL-17 responses in a variety of inflammatory contexts including TB (15–17). IL-17 production following vaccination has been shown to drive a chemokine response that directs a protective T cell response to the lungs (18, 19). Collectively, this work demonstrates that IL-27 activity has important implications in the immune response against Mtb. However, these reports as well as other vaccination studies, have utilized adult animals that do not accurately reproduce the environment in which BCG protection is developed nor when IL-27 is most abundant in circulation.

We previously reported on the influence of IL-27 on neonatal dendritic cells (DCs) (20). IL-27 impaired the ability of neonatal bone marrow-derived DCs (BMDCs) to clear internalized BCG, produce IL-12, and stimulate antigen-specific T cells. This has led us to hypothesize that IL-27 opposes development of protective immune responses following BCG vaccination in neonates that could impact protection against TB disease later in life. To explore this hypothesis, we developed a model of neonatal vaccination with an adult challenge in C57BL/6 (WT) and IL-27 receptor-α-deficient (KO) mice. The latter are unable to respond to IL-27. Here we have shown that WT neonates vaccinated with BCG exhibit variable ability to control Mtb, which we interpreted as recapitulating protection developed in humans. Meanwhile, in the absence of IL-27 signaling, vaccinated neonates exhibit improved control of Mtb burdens and more robust and protective cytokine responses in the lung. This included elevated expression of IFN-γ and IL-17, two canonically important cytokines in defense against TB. These results suggest that blocking IL-27 signaling during neonatal BCG vaccination promotes superior protection from pulmonary TB later in life.

## Methods

### Bacteria preparation

*Mycobacterium bovis*-BCG (Pasteur) and Mtb strain H37Rv were both purchased from the ATCC (Manassas, VA). Mtb strain Erdman smyc’::mCherry that constitutively expresses mCherry and hygromycin was a kind gift of Dr. Joshua Mattila at the University of Pittsburgh. BCG and Mtb stocks were generated from single-colonies on Middlebrook 7H10 agar (Becton Dickinson) supplemented with OADC (Oleic acid, Albumin, Dextrose, Catalase) with or without hygromycin (50 μg/mL) and batch cultured in Middlebrook 7H9 (Becton Dickinson) supplemented with OADC and pimaricin (21.6 µg/ml; Sigma-Aldritch) with or without hygromycin (50 μg/mL). The bacterial stock titer for frozen cultures was established by serial dilution and standard plate counting.

### Neonatal vaccination

BCG was prepared from a pre-titered stock culture. The vaccination dose was pelleted to remove the freezing media, resuspended in PBS with Ca^2+^ and Mg^2+^ at a final volume of 50 ul per mouse and passaged through a 27-gauge needle to dispel clumps. Neonatal pups were vaccinated on days 7 or 8 after birth with 10^3^ CFU BCG in the subscapular region as described previously (20). Males and females were included at approximately equal distributions as litter sizes allowed. As a negative control in all experiments, pups were vaccinated with an equivalent volume of PBS. Mice were rested for 5 weeks after vaccination, at which time serum cytokines were measured or they were aerosol challenged with Mtb.

### Mtb infection

Mice were infected with Mtb by the aerosol route. Mice were placed in mesh baskets at ten or fewer mice per sector within an inhalation chamber (Glas-Col Model A4212). Mtb strain H37Rv was prepared from pre-titered frozen stocks by centrifuging to remove freezing media, suspension in PBS and passage through a 27-gauge needle. A target concentration of 5x10^7^ CFUs/5 ml was applied to the nebulizer for a target delivery of 100 CFUs per mouse. The following exposure settings were used: 15 min warm-up, 35 min nebulize, 30 min cloud decay, 15 min UV exposure (Vacuum Pressure Setting: 60 cubic feet per hour; Comp Air Pressure Setting: 10 cubic feet per hour). Following aerosol infection, the mice were immediately removed from the chamber and housed for 8 weeks. A minimum of 3 otherwise untreated mice were sacrificed at day 1 post-infection to enumerate the initial deposition of bacteria in the lungs. Mice were monitored daily for signs of distress and morbidity. At 8 weeks post-infection, mice were humanely euthanized, and blood and lungs collected for downstream analysis.

### Gene expression of lung cytokines

Lung tissue placed in TriReagent (Sigma) was homogenized using a motorized homogenizer with plastic probes (Omni THb Tissue Homogenizer, Omni International). Chloroform was added per manufacturer recommended protocol, mixed by inversion, and centrifuged at 12,000 x g for 10 minutes at 4°C for phase separation. Aqueous layers were removed and mixed with 70% ethanol. At this point, RNA extraction was completed using the HP Total RNA Extraction kit (Omega Bio-Tek). RNA concentration and purity was measured using a NanoDrop (ThermoFisher) and first-strand cDNA synthesis performed using iScript reagents (Bio-Rad) according to manufacturer recommendation. Real time cycling of reactions that included cDNA from the above preparation diluted in nuclease-free water, gene-specific primer probe sets (Applied Biosystems, Foster City, CA, USA), and iQ™ Supermix (Bio-Rad) was performed in triplicate using a StepOnePlus™ (Applied Biosystems) real time detection system. Gene-specific amplification was normalized to that of *actB* as an internal reference gene and expressed relative to age matched unvaccinated and nonchallenged mice using the formula 2^-ΔΔCt^.

### Cytokine detection by MSD

Cytokines were detected using a U-Plex multiplex electrochemiluminescence assay (MesoScale Discovery). Serum was diluted two-fold in supplied diluent prepared according to manufacturer protocol. All other reagents and incubations were conducted as described in the manufacturer protocol. The prepared plate was analyzed using an MSD Reader and Discovery Workbench (v4.0.13). Protein standards were assayed in parallel with experimental samples.

### Mtb enumeration

Lungs from Mtb-infected mice were placed in 1 mL TRI Reagent^®^ (Sigma). RNA was extracted and cDNA prepared as described above. Mtb was quantified by detection of ESAT6 expression levels in undiluted cDNA. This genetic based approach was necessary to discriminate BCG from Mtb. To enumerate the bacterial burden, a standard curve was generated by adding known amounts of Mtb to age-matched WT lungs and extracting the RNA for measurement of ESAT6 gene expression alongside that of experimental samples as described above and normalized to lung tissue without Mtb. The log_2_ values of the standard curve were plotted against their respective burden to generate a logarithmic curve. The logarithmic equation of the curve (**Supplementary Figure 2**) was used to extrapolate the gene expression value of the experimental samples to determine the Mtb burden in CFUs. To validate analysis of ESAT-6 expression as an approach to enumerate the bacterial burden (**Supplementary Figure 2**), the lungs of Mtb- challenged mice were harvested; the left lobe was placed in TriReagent and processed as described above. The remaining four lobes of right lung were placed in PBS supplemented with Tween^80^, homogenized with a handheld motorized pestle (Kimble Chase, Vineland, NJ), serial diluted, and plated on 7H10 agar supplemented with OADC and hygromycin.

### Statistical analysis

Statistical testing was performed using JMP^®^ Pro version 17 (Cary, NC) or GraphPad Prism version 9 (La Jolla, CA). The threshold for statistical significance was set to alpha = 0.05. Logarithmic transformations were used before performing statistical analyses to reduce the highly skewed nature of the distributions and resulted data well-approximated by normal distributions. Some imputations were necessary; for six mice with an Mtb burden below the level of detection, inputted log burden of zero was applied. The details of the individual tests can be found in the figure legends.

## Results

### IL-27 reduces the efficacy of neonatal BCG vaccination in control of the Mtb burden

**Figure 1:**
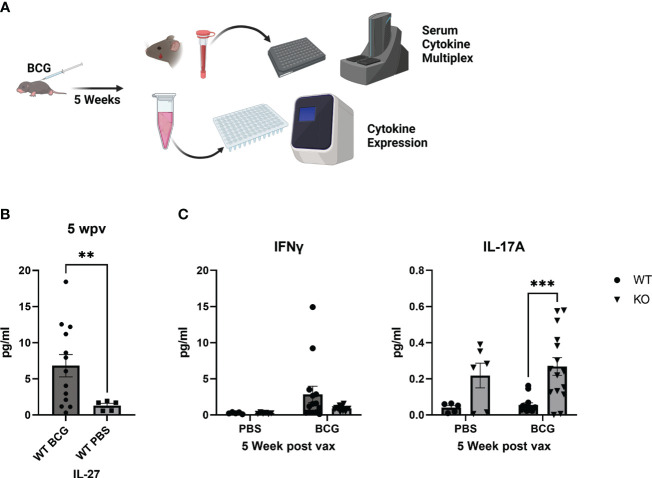
Circulating cytokine responses 5 weeks after neonatal BCG vaccination. **(A)** WT or IL-27Ra-deficient (KO) mice were vaccinated 7 or 8 days after birth with a target dose of 10^3^ BCG/mouse (range: 320-6x10^3^ BCG/mouse) and rested 5 weeks. Serum cytokines were measured by multiplex electrochemiluminescent assay. **(B)** Shown are mean IL-27 concentrations ± SEM for WT PBS control or BCG-vaccinated mice. Each symbol represents an individual mouse. Statistical significance was determined using an unpaired t test with Welch’s correction; ***p=0.0002. **(C)** Shown for WT and KO PBS control and vaccinated mice are mean concentrations ± SEM for IFNγ (left) or IL-17A (right). Each symbol represents an individual mouse. Statistical significance was determined using a two-way ANOVA with Tukey’s multiple comparison test as follow-up; **p=0.0047.

We previously reported the development of a neonatal mouse model of BCG vaccination (20) (**Figure 1A**). The baseline level of serum IL-27 is elevated in neonates compared to older populations (7, 9), and this level continued to rise in the weeks following BCG vaccination [**Figure 1B** (20)]. In the lungs of vaccinated mice, IL-27p28 gene expression was increased compared to nonvaccinated controls and this was very nearly significant (p=0.059, **Supplementary Figure 1A**). EBI3 gene expression in the lung was unchanged and this is consistent with IL-27p28 serving as the rate limiting factor for heterodimer assembly (**Supplementary Figure 1A**). We also previously demonstrated that IL-27 opposed clearance of BCG by BMDCs *in vitro* and in the peripheral tissues of vaccinated mice. Significantly increased numbers of BCG were recovered from the lungs, spleen, and liver 5 weeks after vaccination in WT compared to KO mice that were vaccinated as neonates (20). The increased persistence of BCG was consistent with IL-27 levels at 5 weeks post-vaccination period.

To understand the immunological profile following vaccination and at the time of Mtb challenge, WT or IL-27Rα-KO neonates were vaccinated with BCG or PBS as a control. Five weeks after immunization, a subset of animals was euthanized and serum cytokine levels were measured. WT mice presented with higher IFNγ circulating cytokine than KO counterparts (**Figure 1C**, left). This may be attributed to residual BCG in multiple peripheral tissues continuing to drive an effector response. IFNγ gene expression in the lungs following vaccination was moderately increased in WT mice consistent with this explanation but not significantly different from KO vaccinated mice (**Supplementary Figure 1B**). Meanwhile, vaccinated IL-27Rα-KO mice had greater circulating IL-17 levels than did vaccinated WT animals (**Figure 1C**, right). IL-17 expression, however, was not detected in the lung of either genotype (data not shown). Greater circulating IL-17 levels are in line with an established role for IL-27 in regulation of IL-17; other studies showing animals unresponsive to IL-27 develop more robust Th17 responses (17). This finding is also important as IL-17 has been shown to be a critical cytokine in vaccine-induced protection against Mtb (18, 19, 21), and we predict IL-17 production at the time of challenge would favor a protective response in our neonatal vaccination model.

**Figure 2:**
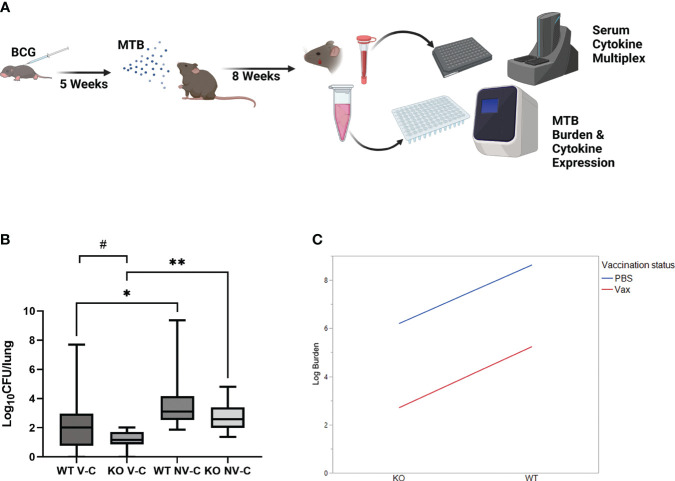
IL-27 signaling during neonatal vaccination compromises control of Mtb burdens in the lungs. **(A)** Shown is a schematic of the vaccination-challenge model. Mice were vaccinated 7 or 8 days after birth with a target dose of 10^3^ BCG/mouse (range: 320-6x10^3^ BCG/mouse) and rested 5 weeks. They were subsequently challenged with a target of 100 CFU/mouse (range: 120-170 CFU/mouse) via the aerosol route. Mice were rested an additional 8 weeks, at which time blood and lungs were collected. **(B)** Mtb burdens were determined by quantification of ESAT6 expression by qPCR relative to a standard curve. Mean log10 CFUs/lung ± SEM for 3 combined experiments with 3-7 mice per experimental group in each experiment are shown. A Welch’s test between vaccinated and non-vaccinated groups demonstrated statistical significance in the 95% confidence interval, *p=0.0425 (WT), **p=0.0004 (KO); a Welch’s test between WT and KO vaccinated animals revealed a trend toward significance,#p=0.051; A two-sided F-test within treatment groups shows significantly less variance in KO vaccinated animals compared to WT counterparts (p<0.0001). The abbreviation V-C indicates vaccinated, challenged; NV-C indicates non-vaccinated, challenged. The same data was then assessed using an interaction plot probing a two-way ANOVA **(C)** and demonstrated significant main effects on improved Mtb burden with either genotype (p=0.015) or vaccination status (p=0.0005).

To determine how the influence of IL-27 signaling during neonatal BCG vaccination contributes to protection against Mtb infection, WT and KO mice were challenged with Mtb by the aerosol route five weeks post-vaccination. For the three collective experiments the actual delivery to the lungs at day 1 post-infection ranged from 120-170. Mice were rested for eight weeks, at which time blood was collected by submandibular bleeding and lungs harvested after humane euthanasia (**Figure 2A**). Bacterial burdens were enumerated by real-time PCR-based measurement of ESAT6 expression to discriminate persistent BCG from Mtb in line with TB diagnostic approaches; colonies of either bacteria cannot be accurately differentiated on plates. Experimental samples were compared to known CFUs spiked into lung tissue to generate a standard curve of amplification (**Supplementary Figure 2A**). This method was separately validated by challenge with a hygromycin-resistant strain of Mtb in which the ESAT6 expression in the left lung correlated positively with standard plate counts of CFUs in the right lung (**Supplementary Figure 2B**). WT mice vaccinated with BCG as neonates exhibited significantly improved control of Mtb compared to non-vaccinated animals but recapitulated the high range of variable protection that is observed in humans (**Figure 2B**). KO mice also exhibited significant protection from vaccination (**Figure 2B**). The variation in protection within the KO vaccinated mice was significantly reduced and different from that of the WT vaccinated mice as determined by an F test (p<0.0001). Compared to WT vaccinated and Mtb challenged mice, the improved reduction in lung bacterial burdens measured in KO vaccinated mice was nearly statistically significant, only narrowly outside the 95% confidence interval (p=0.051). In summary, we observed significant main effects of genotype (p=0.0150) and vaccination (p=0.0005), indicating that the host response in the absence of IL-27 signaling and with vaccination offers significant, albeit independent, protection against Mtb burden (**Figure 2C**).

### Abrogation of IL-27 signaling during neonatal vaccination increases IFNγ in the periphery following Mtb challenge

**Figure 3:**
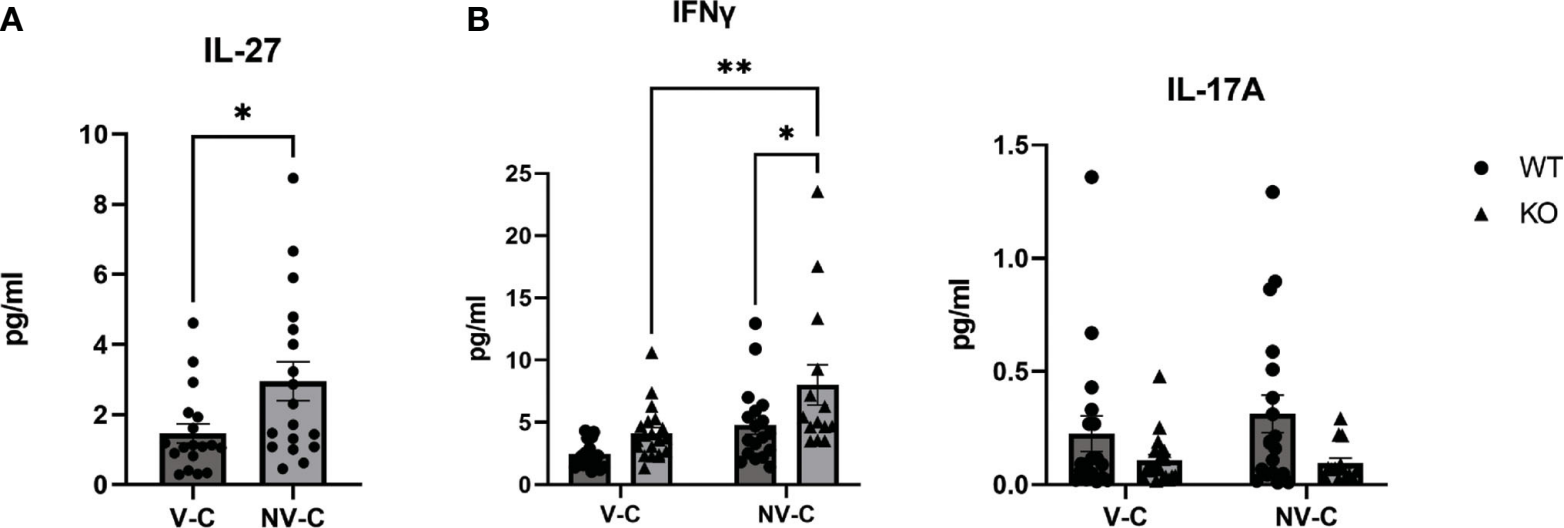
The circulating cytokine profile following neonatal BCG vaccination and Mtb aerosol challenge. Mice were vaccinated as neonates with BCG or PBS as previously described and then aerosol challenged with Mtb for eight weeks. Prior to euthanization, mice were anesthetized for submandibular bleeding to determine serum cytokine concentration by multiplex electrochemiluminescent assay. Three combined experiments with 3-7 mice per experimental group are shown. **(A)** The mean concentration of IL-27 ± SEM in WT mice treated as indicated is shown. Statistical significance in the 95% confidence interval was determined using Mann-Whitney t test; *p=0.01. Mean results ± SEM of **(B)** IFNγ and IL-17A from WT and KO mice treated as indicated are shown. Statistical significance in the 95% confidence interval was determined using a two-way ANOVA followed by Tukey’s multiple comparison test; *p=0.042, **p=0.0074. The abbreviation V-C indicates vaccinated, challenged; NV-C indicates non-vaccinated, challenged.

As there were reduced bacterial burdens in the absence of IL-27 signaling following neonatal vaccination, we examined the peripheral immune responses for correlates of protection. Analysis of serum cytokines 8 weeks post-challenge of control and vaccinated mice indicated that infection by Mtb induced production of IL-27, in addition to inflammatory cytokines IL-17 and IFNγ (**Figure 3**). In the WT mice, IL-27 production was significantly greater in non-vaccinated, challenged animals (**Figure 3A**). This increased IL-27 production could be the result of and/or contribute to the poor and highly variable control of Mtb reported in **Figure 2**. However, it is important to note that a direct correlation between levels of IL-27 in the serum and bacterial burdens in the lung was not demonstrated (**Supplementary Figure 3**). IFNγ levels in KO animals were significantly higher in non-vaccinated, challenged animals compared to WT counterparts as well as vaccinated, challenged KO animals. (**Figure 3B**, left). Meanwhile, circulating IL-17 responses were not greatly elevated in any of our observed groups (**Figure 3B**, right). The low concentrations of this cytokine detected systemically may suggest the influence of IL-17 in the immune response acts more local in the lungs following Mtb challenge. Collectively, this suggests that IFNγ and IL-27p28 are important cytokines in the immune response after Mtb challenge, and mice unresponsive to IL-27 are more poised to have increased levels of IFNγ in the periphery.

### Loss of IL-27 signaling during neonatal vaccination alters the cytokine profile in the lungs following Mtb challenge

**Figure 4:**
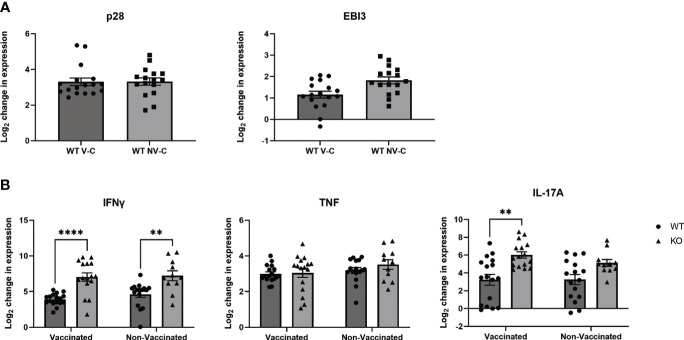
The lung cytokine profile following neonatal BCG vaccination and Mtb aerosol challenge. WT and KO mice were vaccinated as neonates with BCG or PBS as previously described, then aerosol challenged with Mtb or left uninfected for 8 weeks. Gene expression analysis was performed by real-time PCR and the data expressed as the log2 change relative to age-matched uninfected mice using the formula 2-^ΔΔCT^. Three combined experiments with 3-7 mice per experimental group are shown. **(A)** The mean change in expression ± SEM in WT mice treated as indicated for the IL-27 subunits p28 and EBI3 is shown. “V-C” is “vaccinated, challenged”, “NV-C” is “Non-vaccinated, challenged”. **(B)** The mean change in expression ± SEM for IFNγ, TNF, and IL-17A in the lungs of WT and KO BCG or PBS-vaccinated and Mtb challenged animals. **(A, B)** Statistical significance in the 95% confidence interval was assessed using a two-way ANOVA followed by Tukey’s Multiple comparison; **p=0.001 (IL-17) or 0.0035 (IFNγ), ****<0.0001.

To examine the immunological profile in the lungs that accompanied improved consistency of bacterial clearance in the absence of IL-27 signaling, we measured the expression of cytokines known to be important during TB. Since we utilized a molecular biology approach to detect Mtb, we were able to investigate gene expression from the same lungs that were used to quantify bacterial burdens for maximal correlation. In IL-27 responsive WT animals, similar increases in expression of p28 compared to naïve animals were observed between both challenge groups (**Figure 4A** left). EBI3, however, was slightly increased in non-vaccinated, challenged animals compared to vaccinated counterparts (**Figure 4A**). We observed significantly greater IFNγ gene expression in KO Mtb-challenged animals compared to WT in both vaccinated and non-vaccinated groups (**Figure 4B**, left). Although we observed improved IFNγ responses in the lungs of our KO animals, TNF gene expression was consistent across all groups, indicating it was not impacted by the loss of IL-27 signaling (**Figure 4B**, middle). However, vaccinated KO mice did have significantly increased expression of IL-17 compared to WT counterparts (**Figure 4B**, right). Loss of IL-27 signaling has been shown previously to increase expression of IL-17 (15–17).

### TNF expression combined with low IFNγ expression is consistent with variable control of Mtb

**Figure 5:**
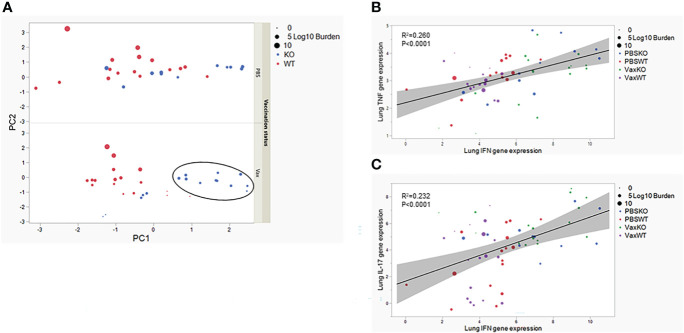
Multivariate analysis of inflammation and control of Mtb in the lungs of mice vaccinated as neonates. **(A)** A principal component analysis is displayed based on vaccination status, where PC1 is weighted on inflammatory gene expression and PC2 weights on Mtb burden. The black oval highlights the grouping of KO animals weighted on PC1. **(B)** A correlation plot of TNF and IFNγ expression in the lung demonstrates the grouping of WT animals by red and purple symbols (left middle). Individual symbols are labeled according to WT or KO genotype and sized according to bacterial burden magnitude. Statistical significance assessed by Pearson correlation coefficient, p<0.0001 **(C)** A correlation plot of IL-17 and IFNγ expression levels in the lung demonstrates the grouping of KO animals by blue and green symbols (upper right). Individual symbols are labeled according to WT or KO genotype and sized according to bacterial burden magnitude. Statistical significance assessed by Pearson correlation coefficient, p<0.0001.

The pattern of individual cytokine expression in the lungs or periphery does not alone explain more consistent control of Mtb in KO mice vaccinated as neonates. None of the measured cytokines individually has a direct correlation with bacterial burdens (data not shown). Therefore, we utilized all the continuous measures in the lung at the site of infection in a multivariate principal component analysis (PCA) analysis. The first PC (PC1) weighted the gene expression of IFNγ, TNF, and IL-17 heavily. The second PC (PC2) weighted the Mtb burdens heavily. When PC1 and PC2 are plotted for each mouse by vaccination status, the KO mice vaccinated as neonates with BCG exhibit sharper grouping weighted as above average on PC1 which is the combined expression of IFNγ, TNF, and IL-17 (**Figure 5A**, black oval). In contrast the vaccinated WT mice are more centered on each PC (**Figure 5A**). This suggests a multi-functional cytokine response in the lungs could be attributed to more consistent control of Mtb in the absence of IL-27 signaling following neonatal vaccination. To further explore this possibility, we performed a correlation matrix with all the measurements included in **Figures 2**, **4** except for IL-27. This analysis focused on the WT and KO mice vaccinated with BCG or given PBS as neonates and challenged with Mtb and revealed a significant correlation (p<0.0001) of TNF and IFNγ gene expression in the lungs. As these are both proinflammatory cytokines, this association is not unexpected. However, when we compared the TNF expression plotted with IFNγ expression, most of the WT vaccinated mice grouped together regardless of treatment (**Figure 5B**). Consistent with these observations, the variation of TNF and IFNγ gene expression was statistically significant between WT and KO animals (p=0.0034 and p=0.0075, respectively). Similarly, IL-17 gene expression and IFNγ gene expression in the lungs correlate positively (p<0.0001). Here, KO animals consistently showed a correlation with higher IFNγ and IL-17 expression (**Figure 5C**). Consistent with these observations, the variation of IL-17 expression was greater and statistically significant (p=0.011). We did not observe a positive or negative correlation in either assessment of burdens with expression of cytokines. Collectively, these data suggest that overall consistent and strong TNF expression combined with lower IL-17 and IFNγ expression fails to consistently control Mtb in WT animals. Moreover, though the amount of an individual cytokine does not predict outcomes following Mtb infection, in the absence of IL-27, the increase in a combined cytokine response is consistent with lower bacterial burdens. The combination of cytokines and respective levels is also likely important to this protection, as even a significant increase in any one cytokine is not associated with improved control of bacteria (data not shown).

## Discussion

In the present study, we sought to understand the role of IL-27 during neonatal BCG vaccination and its influence on protection developed against Mtb challenge. An emphasis of our study design was to model human vaccination and challenge as best possible from a timeline and developmental perspective. Although it is difficult to accurately represent every aspect of human disease in animal models, due to the more rapid development of mice, it is possible to mimic the human scenario. That is one in which vaccination occurs in the neonatal period and robust exposure to pulmonary TB occurs in adolescence to adulthood when the benefits of BCG vaccination have been shown to wane as consistent long-term protection is not observed (3). We readily acknowledge that this model does not incorporate broad genetic diversity. First, we investigated the existing immune response five-weeks after vaccination, the time at which we challenged mice. As we have previously reported (20) but also important to establish in the challenged cohorts, we saw elevated IL-27 in vaccinated WT animals. Since IL-27 continues to rise through 5 weeks post-vaccination, it is likely that it shapes the immune response to BCG.

**Figure 6:**
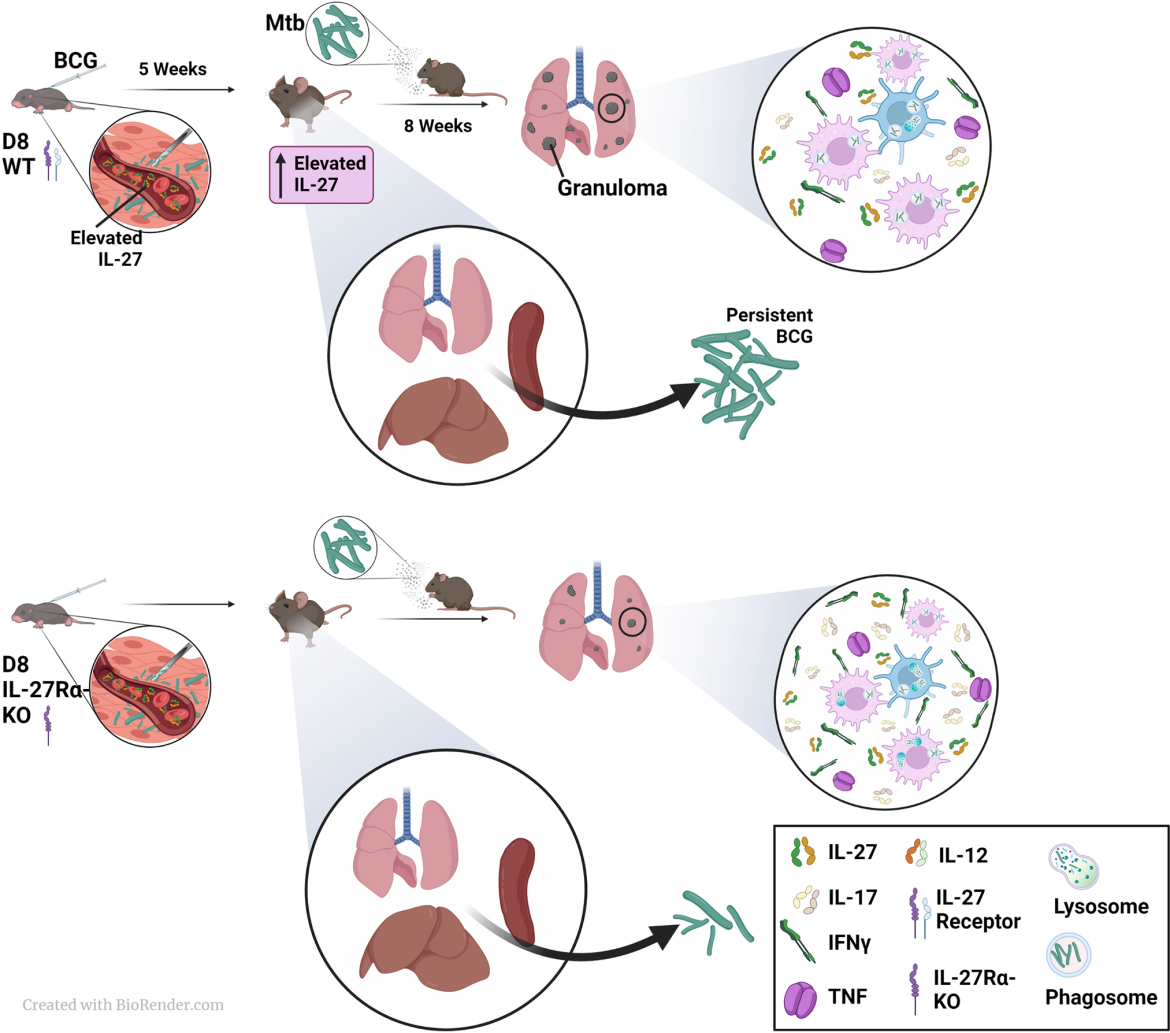
A model to summarize the involvement of IL-27 in neonatal vaccination and adult Mtb challenge. WT or IL-27Rα-KO animals were vaccinated with BCG as neonates and rested 5 weeks. During this time IL-27 levels continue to rise and can shape immune responses in the WT mice that can respond. Mice unresponsive to IL-27 clear BCG from peripheral tissues more efficiently than WT mice, a finding we reported previously. Following challenge with Mtb, mice were rested for 8 weeks at which time bacterial burden and cytokine responses were assessed. Mice unable to respond to IL-27 also controlled Mtb more efficiently in their lungs than WT counterparts, and this was coupled with improved expression of IL-17 and IFNγ in lungs. Coupled with our previous findings on the DC response to BCG in the absence of IL-27 (20) and other works showing its effects on macrophages, we hypothesize IL-27 reduces the phagocytic potential of cells, alters the inflammatory cytokine milieu, and limits the protective immunity against Mtb.

In our prior work, we demonstrated that clearance of BCG is impaired in neonatal DCs in response to IL-27 (20). In addition, IL-12 production was reduced in response to BCG and the stimulation of IFNγ by T cells from BCG-vaccinated mice was limited compared with DCs from IL-27Rα-deficient mice (20). Extending those findings to the current *in vivo* model (**Figure 6**), we did not observe significant increases in circulating IFNγ in either genotype five-weeks after vaccination, but we did see statistically greater IL-17 in the serum of our vaccinated IL-27Rα-KO animals compared to WT. Previously we showed that neonatally-vaccinated WT mice maintain significantly greater BCG in their peripheral tissues compared to KO animals (20). We hypothesized this may drive a continual effector CD4^+^ T cell immune response as opposed to developing long-term central memory populations, and with the observed elevation of IL-27, a less effective inflammatory response that would not provide protective immunity. Therefore, we evaluated how the improved clearance of BCG and increased IL-17 production in our KO-vaccinated animals would impact responses against Mtb challenge. To do so in a manner that did not count persistent BCG as Mtb post challenge, we needed to employ an approach that discriminated the bacteria. The gold standard IFN-γ release assay (IGRA) to diagnose TB exposure and infection incorporates ESAT6 and CFP-10 that are not expressed by BCG. As such, we adopted similar rationale by measuring ESAT6 gene expression as an indicator of Mtb viability. While we cannot exclude some differences in ESAT6 expression with various *in vivo* conditions, it is our expectation that the *in vivo* environment is not drastically different between WT and KO mice such that the absence of a single host gene would dictate dissimilar ESAT6 expression. In line with this, we performed separate experiments that validate a significant positive correlation of ESAT-6 expression in the left lung with standard plate counts on media supplemented with hygromycin to select for Mtb only (**Supplementary Figure 2**). We observed an overall decrease in Mtb burden by detection of ESAT6 expression in the lungs of both vaccinated mice WT and IL-27Rα-KO genotypes compared to non-vaccinated animals (**Figure 2**). Furthermore, while KO mice vaccinated as neonates consistently controlled Mtb, with very low bacterial burdens detected in their lungs 8 weeks after challenge, WT mice vaccinated as neonates exhibited significant heterogeneity in their ability to elicit a protective response (**Figure 2B**). This resembles what is observed in humans whereby BCG has 0-85% protective efficacy (3). Importantly our model involves inbred mice that do not recapitulate the genetic diversity in humans, however, as it has not been examined in an experimental model, heterogeneity in the immune response of neonates as a result of the immaturity of the immune system may translate to variability in protection. Furthermore, while there are countless studies in which mice and even NHPs are vaccinated with BCG as adults, most do not report this extensive variability in protection from Mtb challenge (22–25). This underscores the novelty of our model and the importance of neonatal vaccination to better recapitulate the extensive variability of protection by BCG in humans.

We observed that neonatal vaccination and absence of IL-27 signaling significantly contributed to Mtb clearance after challenge, but these two variables were independent, with the rate of improved protection observed between vaccinated WT and vaccinated KO mice comparable to their respective non-vaccinated counterparts (**Figure 2C**). Interestingly, though, the amount of circulating IL-27 did not correlate with Mtb burden. Additionally, despite significant differences in circulating IL-27 between vaccinated and non-vaccinated animals, lung gene expression of the two subunits was comparable between treatment groups. Considering the higher levels of IL-27 in the serum of non-vaccinated mice, it is unclear if the rate of gene expression peaked prior to 8 weeks post-challenge, EBI3 is the limiting factor in assembly of the secreted heterodimer, there is post-transcriptional regulation of IL-27 subunits that impacts protein production between the two groups, or differences in IL-27 expression at other peripheral sites contributes to the circulating cytokine differences. Paired with findings in **Figure 2**, this would suggest that IL-27 is detrimental in the control of Mtb but the absolute level does not dictate the magnitude of dysregulated host protection. Overall, the absence of IL-27 signaling significantly improved response against Mtb, as did vaccination in either genotype, with KO animals consistently controlling bacterial burden more efficiently than WT mice. This follows trends previously observed of IL-27 unresponsive animals exhibiting an improved ability to control Mtb after infection (10, 11, 13, 17). However, we utilized a novel approach here in which we replicated the age of BCG vaccination in humans by immunizing mice in the neonatal period.

To further improve vaccination for protection against TB it is necessary to identify the constituents of protective immunity. IFNγ is canonically associated with protective immune responses to Mtb because of its role in activating macrophages. Previously IFNγ expression was shown to be decreased in Mtb-infected IL-27Rα KO mouse lungs (10). However, other studies suggest this deficiency is only apparent early during infection, as IFNγ responses later in infection are equal or even greater in IL-27Rα KO mice (11, 26). Here, we observed elevated circulating IFNγ in KO animals which was significantly greater in vaccinated KO animals than their non-vaccinated counterparts as well as significantly increased above vaccinated WT mice. However, we observed vaccinated animals produced less IFNγ compared to their non-vaccinated counterparts in both genotypes. This may be reflective of reduced Mtb burdens and an indication of a contracting immune response following improved bacterial control. It is also possible that the lower systemic cytokine level is reflective of a more appropriate level of inflammation. In the lung, KO animals had elevated IFNγ expression compared to WT animals in both the vaccinated and non-vaccinated groups (**Figure 4**). Interestingly, the degree of gene expression within each genotype was approximately the same regardless of vaccination status. This may imply that IFNγ expression has the potential to be induced up to a certain point within each genotype in the response to Mtb in the lung. Although this finding is consistent with IL-27 regulation of inflammation, it does suggest IFNγ expression alone is not correlated with the improved bacterial clearance in vaccinated mice, as the level of gene expression is the same between vaccinated and non-vaccinated KO mice.

Along with the role of IL-27 as a regulator of inflammatory responses, IL-27 has been demonstrated to inhibit development of Th17 immune responses (15–17). Surprisingly, though we observed increased IL-17 in the serum from vaccinated IL-27Rα-KO five weeks after vaccination (**Figure 1**), we did not observe elevated circulating IL-17 following eight weeks of Mtb challenge. IL-17 has an established role in recruitment of CD4^+^ T cells to the lung following vaccination and promotion of granuloma development (19, 27). This includes accumulation of multifunctional T cells in the lung (17). Thus, increased IL-17 levels observed in KO mice vaccinated as neonates at the time of Mtb challenge is likely to have directed a protective response to the lungs and subsequently receded in the periphery. While serum IL-17 levels are low overall, it is notable that they were higher in WT mice following challenge, despite the increased expression of this cytokine in the lungs of KO animals (**Figures 3**, **4**). One explanation for this could be that this systemic IL-17 is produced by neutrophils, which have been shown to be contributors to IL-17 during Mtb infection (28). However, the gene expression of IL-17 in the lungs was higher in KO animals, and significantly greater in those which were previously vaccinated (**Figure 4**). This suggests an overall improved local Mtb response in the absence of IL-27. Indeed, IL-17^+^IFNγ^+^ CD4^+^ T cells have previously been shown to preferentially localize to the lungs of infected non-human primates and correlate with reduced Mtb burden (29).

Although there are differences in IL-17 and IFNγ expression patterns between WT and KO mice vaccinated as neonates, neither cytokine independently correlated with bacterial burdens. However, when we evaluate the combined inflammatory gene profile and bacterial burdens by multivariate analysis, we see a trend emerge indicating improved control of bacteria with an overall greater inflammatory response in KO mice vaccinated as neonates (**Figure 5**). Our data demonstrate that in a correlation curve of Th1 cytokines, WT mice cluster towards a different balance of IFN-g and TNF that is consistent with higher bacterial burdens. This imbalance may not promote effective antimycobacterial activity in macrophages. A similar trend was demonstrated in the correlation analysis of IFNγ and IL-17. This suggests to us that, while the absence of IL-27 signaling clearly improves Mtb clearance, these effects are not mediated through a change in any single inflammatory Th1 or Th17 cytokine alone but is rather a total shift towards a combined inflammatory response. This is in line with prior work underscoring the importance of IL-17, TNF, and IFNγ in the control of Mtb (19, 30, 31) as well as the importance of multi-functional T cell responses (13, 17, 32).

Our data supports that neonatal BCG vaccination in the absence of IL-27 signaling better primes the immune system for a more rapid response to Mtb. We show here that in addition to more consistent control of Mtb, IL-27Rα KO mice also produce a more robust and balanced inflammatory response, which correlation analyses indicate contributes to the improved bacterial control. We suspect an additional element of this improved immune response could involve a more diverse T cell response and this is an aspect of ongoing investigation. In summary, as significant strength of this work is the combined modeling of neonatal vaccination with adult pulmonary challenge. In addition, the genetic approach to enumerating the Mtb burden allowed for direct correlation of cytokine expression data in the same piece of tissue. The former has important translational implications as it is consistent with trends in human vaccination and the duration of BCG protection in endemic TB regions. Efforts to improve TB vaccination should consider vaccination during the neonatal period and the implications of IL-27 signaling in early life immunity.

## Data availability statement

The raw data supporting the conclusions of this article will be made available by the authors, without undue reservation.

## Ethics statement

The animal study was approved by West Virginia University Institutional Animal Care and Use Committee. The study was conducted in accordance with the local legislation and institutional requirements.

## Author contributions

SDB performed and designed experiments, analyzed and interpreted data, and wrote the manuscript. AD performed and designed experiments. SB performed experiments. JP and KR analyzed and interpreted data and contributed to the manuscript. CR supervised all research, designed and performed experiments, analyzed and interpreted results, and wrote the manuscript. All authors contributed to the article and approved the submitted version.

## Supplementary material

Supplementary Figure 1 The lung cytokine profile following neonatal BCG vaccination. WT or IL-27Ra-deficient (KO) mice were vaccinated 7 or 8 days after birth with a target dose of 10^3^ BCG/mouse (range: 320-6x10^3^ BCG/mouse) and rested 5 weeks. Gene expression analysis of lung tissue was performed by real-time PCR and the data expressed as the log2 change relative to age-matched non-vaccinated mice using the formula 2-^ΔΔCT^. (**A**) The mean change in expression ± SEM in WT mice treated as indicated for the IL-27 subunits p28 and EBI3 is shown. Statistical significance was determined using an unpaired t test with Welch’s correction. The p value is shown for the comparison to the non-vaccinated controls for each gene separately. (**B**) The mean change in expression ± SEM relative to the non-vaccinated controls for IFNγ in each genotype is shown. Statistical significance was determined using an unpaired t test with Welch’s correction to compare the change in vaccinated mice for the respective genotypes.

Supplementary Figure 2 The standard curve and validation for ESAT6-based bacterial enumeration. **(A)** To enumerate the bacterial burden, a standard curve was generated by adding known amounts of Mtb CFUs to age-matched WT lungs and extracting the RNA for measurement of ESAT6 gene expression alongside that of experimental samples as described above and normalized to lung tissue without Mtb. The log_2_ values of the standard curve were plotted against their respective burden to generate the logarithmic curve. The logarithmic equation of the curve was used to extrapolate the gene expression value of the experimental samples to determine the Mtb burden in CFUs. **(B, C)** Control and BCG-vaccinated WT and KO mice were challenged with hygromycin-resistant Mtb that constitutively express mCherry providing dual traits that differentiate from BCG. Following 8 weeks of challenge, the left lung was committed to RNA isolation and measurement of ESAT6 expression. The right lung of the same mouse was homogenized for serial dilution and plating on media supplemented with hygromycin. Correlation plots for separate experiments performed at separate times in which symbols correspond to individual mice are shown.

Supplementary Figure 3 IL-27 levels in the periphery do not correlate with control of Mtb in the lungs of mice vaccinated as neonates. Correlation plots of IL-27 serum levels and log10 burdens of Mtb in the lung. Individual symbols correspond to individual mice. The R^2^ and p values are indicated for the unvaccinated and Mtb challenged mice **(A)**, the BCG-vaccinated and challenged mice **(B)**, or the combined groups **(C)** respectively.

## References

[1] Organization WH. Global Tuberculosis Report 2022 (2022). Available at: https://www.who.int/teams/global-tuberculosis-programme/tb-reports/global-tuberculosis-report-2022/tb-disease-burden/2-1-tb-incidence.

[2] Barcat JA Kantor IN Ritacco V. One hundred years of BCG vaccine. Med (B Aires) (2021) 81(6):1007–14. 34875601

[3] Mangtani P Abubakar I Ariti C Beynon R Pimpin L Fine PE. Protection by BCG vaccine against tuberculosis: a systematic review of randomized controlled trials. Clin Infect Dis (2014) 58(4):470–80. doi: 10.1093/cid/cit790 24336911

[4] Snow KJ Sismanidis C Denholm J Sawyer Susan M Graham SM. The incidence of tuberculosis among adolescents and young adults: a global estimate. Eur Respir J (2018) 51(2):1702352. doi: 10.1183/13993003.02352-2017 29467206

[5] Prevention CfDCa. Child and Adolescent Immunization Schedule. Center for Disease Control and Prevention (2022). Available at: https://www.cdc.gov/vaccines/schedules/hcp/imz/child-adolescent.html.

[6] Tsafaras GP Ntontsi P Xanthou G. Advantages and Limitations of the neonatal immune system. Front Pediatr (2020) 8:5–. doi: 10.3389/fped.2020.00005 PMC6997472 32047730

[7] Kraft JD Horzempa J Davis C Jung JY Pena MM Robinson CM. Neonatal macrophages express elevated levels of interleukin-27 that oppose immune responses. Immunology (2013) 139(4):484–93. doi: 10.1111/imm.12095 PMC3719065 23464355

[8] Hunter HYaCA. The immunobiology of interleukin-27. Annu Rev Immunol (2015) 33:417–43. doi: 10.1146/annurev-immunol-032414-112134 25861977

[9] Gleave Parson M Grimmett J Vance JK Witt MR Seman BG Rawson TW. Murine myeloid-derived suppressor cells are a source of elevated levels of interleukin-27 in early life and compromise control of bacterial infection. Immunol Cell Biol (2019) 97(5):445–56. doi: 10.1111/imcb.12224 PMC6536317 30575117

[10] Pearl JE Khader SA Solache A Gilmartin L Ghilardi N deSauvage F. IL-27 signaling compromises control of bacterial growth in mycobacteria-infected mice. J Immunol (2004) 173(12):7490–6. doi: 10.4049/jimmunol.173.12.7490 15585875

[11] Holscher C Holscher A Ruckerl D Yoshimoto T Yoshida H Mak T. The IL-27 receptor chain WSX-1 differentially regulates antibacterial immunity and survival during experimental tuberculosis. J Immunol (2005) 174(6):3534–44. doi: 10.4049/jimmunol.174.6.3534 15749890

[12] Robinson CM Nau GJ. Interleukin-12 and interleukin-27 regulate macrophage control of Mycobacterium tuberculosis. J Infect Dis (2008) 198(3):359–66. doi: 10.1086/589774 PMC2761687 18557702

[13] Torrado E Fountain JJ Liao M Tighe M Reiley WW Lai RP. Interleukin 27R regulates CD4+ T cell phenotype and impacts protective immunity during Mycobacterium tuberculosis infection. J Exp Med (2015) 212(9):1449–63. doi: 10.1084/jem.20141520 PMC4548054 26282876

[14] Villarino A Hibbert L Lieberman L Wilson E Mak T Yoshida H. The IL-27R (WSX-1) is required to suppress T cell hyperactivity during infection. Immunity (2003) 19:645–55. doi: 10.1016/S1074-7613(03)00300-5 14614852

[15] Stumhofer JS Laurence A Wilson EH Huang E Tato CM Johnson LM. Interleukin 27 negatively regulates the development of interleukin 17-producing T helper cells during chronic inflammation of the central nervous system. Nat Immunol (2006) 7(9):937–45. doi: 10.1038/ni1376 16906166

[16] Diveu C McGeachy MJ Boniface K Stumhofer JS Sathe M Joyce-Shaikh B. IL-27 blocks RORc expression to inhibit lineage commitment of Th17 cells. J Immunol (2009) 182(9):5748–56. doi: 10.4049/jimmunol.0801162 19380822

[17] Erdmann H Behrends J Ritter K Holscher A Volz J Rosenkrands I. The increased protection and pathology in Mycobacterium tuberculosis-infected IL-27R-alpha-deficient mice is supported by IL-17A and is associated with the IL-17A-induced expansion of multifunctional T cells. Mucosal Immunol (2018) 11(4):1168–80. doi: 10.1038/s41385-018-0026-3 29728641

[18] Gopal R Lin Y Obermajer N Slight S Nuthalapati N Ahmed M. IL-23-dependent IL-17 drives Th1-cell responses following Mycobacterium bovis BCG vaccination. Eur J Immunol (2012) 42(2):364–73. doi: 10.1002/eji.201141569 PMC3490408 22101830

[19] Khader SA Bell GK Pearl JE Fountain JJ Rangel-Moreno J Cilley GE. IL-23 and IL-17 in the establishment of protective pulmonary CD4+ T cell responses after vaccination and during Mycobacterium tuberculosis challenge. Nat Immunol (2007) 8(4):369–77. doi: 10.1038/ni1449 17351619

[20] Bradford SD Witt MR Povroznik JM Robinson CM. Interleukin-27 impairs BCG antigen clearance and T cell stimulatory potential by neonatal dendritic cells. Curr Res Microb Sci (2023) 4:100176. doi: 10.1016/j.crmicr.2022.100176 36530844 PMC9747568

[21] Gopal R Rangel-Moreno J Slight S Lin Y Nawar HF Fallert Junecko BA. Interleukin-17-dependent CXCL13 mediates mucosal vaccine-induced immunity against tuberculosis. Mucosal Immunol (2013) 6(5):972–84. doi: 10.1038/mi.2012.135 PMC3732523 23299616

[22] Darrah PA Zeppa JJ Maiello P Hackney JA Wadsworth MH Hughes TK. Prevention of tuberculosis in macaques after intravenous BCG immunization. Nature (2020) 577(7788):95–102. doi: 10.1038/s41586-019-1817-8 31894150 PMC7015856

[23] Plumlee CR Barrett HW Shao DE Lien KA Cross LM Cohen SB. Assessing vaccine-mediated protection in an ultra-low dose *Mycobacterium tuberculosis* murine model. PLoS Pathog (2023) 19(11):e1011825. doi: 10.1371/journal.ppat.1011825 38011264 PMC10703413

[24] Smith CM Proulx MK Olive AJ Laddy D Mishra BB Moss C. Tuberculosis susceptibility and vaccine protection are independently controlled by host genotype. mBio (2016) 7(5):e01516-16. doi: 10.1128/mBio.01516-16 PMC5030360 27651361

[25] Ferreira CM Micheli C Barreira-Silva P Barbosa AM Resende M Vilanova M. IL-10 overexpression after BCG vaccination does not impair control of mycobacterium tuberculosis infection. Front Immunol (2022) 13:946181. doi: 10.3389/fimmu.2022.946181 35935958 PMC9353026

[26] Yoshida H Hamano S Senaldi G Covey T Faggioni R Mu S. WSX-1 is required for the initiation of th1 responses and resistance to L. major Infection. Immunity (2001) 15(4):569–78. doi: 10.1016/s1074-7613(01)00206-0 11672539

[27] Yahagi A Umemura M Tamura T Kariyone A Begum MD Kawakami K. Suppressed induction of mycobacterial antigen-specific Th1-type CD4+ T cells in the lung after pulmonary mycobacterial infection. Int Immunol (2010) 22(4):307–18. doi: 10.1093/intimm/dxq010 20167585

[28] Hu S He W Du X Yang J Wen Q Zhong XP. IL-17 Production of Neutrophils Enhances Antibacteria Ability but Promotes Arthritis Development During Mycobacterium tuberculosis Infection. EBioMedicine (2017) 23:88–99. doi: 10.1016/j.ebiom.2017.08.001 28821374 PMC5605331

[29] Shanmugasundaram U Bucsan AN Ganatra SR Ibegbu C Quezada M Blair RV. Pulmonary Mycobacterium tuberculosis control associates with CXCR3- and CCR6-expressing antigen-specific Th1 and Th17 cell recruitment. JCI Insight (2020) 5(14):e137858. doi: 10.1172/jci.insight.137858 32554933 PMC7453885

[30] Caruso AM Serbina N Klein E Triebold K Bloom BR Flynn JL. Mice deficient in CD4 T cells have only transiently diminished levels of IFN-γ, yet succumb to tuberculosis1. J Immunol (1999) 162(9):5407–16. doi: 10.4049/jimmunol.162.9.5407 10228018

[31] Scott Algood HM Lin PL Flynn JL. Tumor necrosis factor and chemokine interactions in the formation and maintenance of granulomas in tuberculosis. Clin Infect Dis (2005) 41(Supplement_3):S189–S93. doi: 10.1086/429994 15983898

[32] Derrick SC Yabe IM Yang A Morris SL. Vaccine-induced anti-tuberculosis protective immunity in mice correlates with the magnitude and quality of multifunctional CD4 T cells. Vaccine (2011) 29(16):2902–9. doi: 10.1016/j.vaccine.2011.02.010 21338678

