# Supplementary figures and images for: IL-27 alters inflammatory cytokine expression and limits protective immunity against Mycobacterium tuberculosis in a neonatal BCG vaccination model
Source: Front Immunol. 2024 Feb 8;15:1217098. doi: 10.3389/fimmu.2024.1217098 (PMC10881868; doi:10.3389/fimmu.2024.1217098)

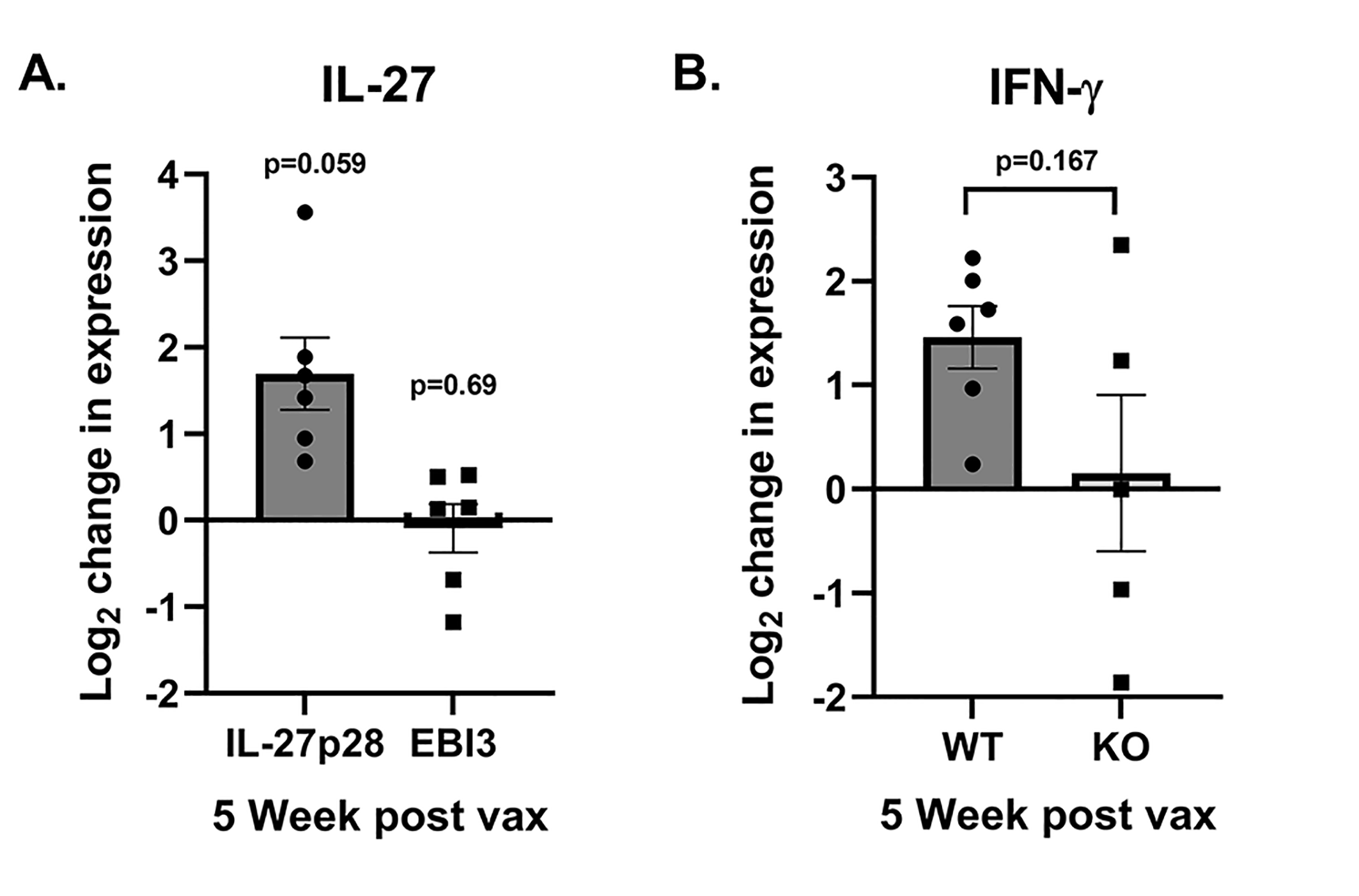

Supplement: Supplementary Figure 1 — The lung cytokine profile following neonatal BCG vaccination. WT or IL-27Ra-deficient (KO) mice were vaccinated 7 or 8 days after birth with a target dose of 103 BCG/mouse (range: 320-6x10^3^ BCG/mouse) and rested 5 weeks. Gene expression analysis of lung tissue was performed by real-time PCR and the data expressed as the log2 change relative to age-matched non-vaccinated mice using the formula 2-ΔΔCT. (A) The mean change in expression ± SEM in WT mice treated as indicated for the IL-27 subunits p28 and EBI3 is shown. Statistical significance was determined using an unpaired t test with Welch’s correction. The p value is shown for the comparison to the non-vaccinated controls for each gene separately. (B) The mean change in expression ± SEM relative to the non-vaccinated controls for IFNγ in each genotype is shown. Statistical significance was determined using an unpaired t test with Welch’s correction to compare the change in vaccinated mice for the respective genotypes. [file Image_1.tif]

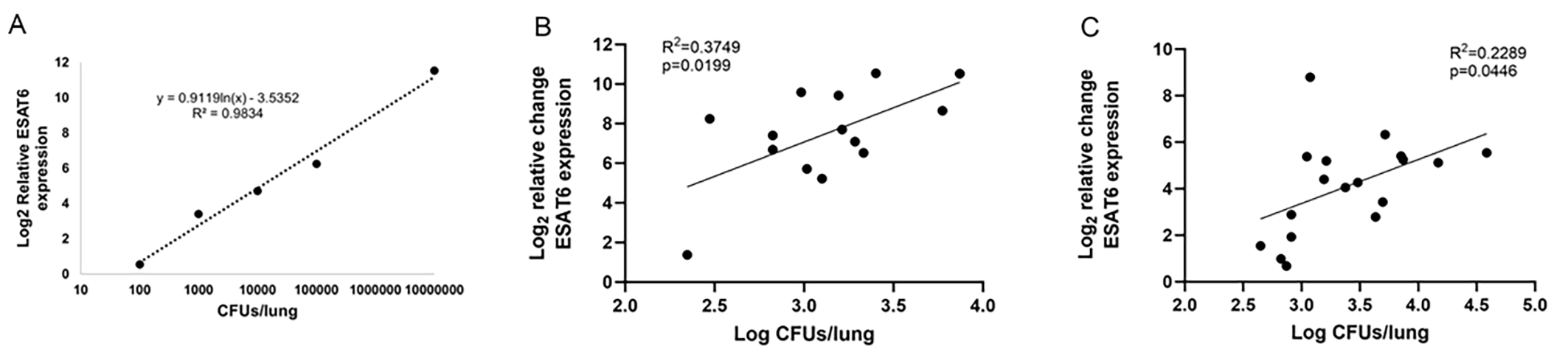

Supplement: Supplementary Figure 2 — The standard curve and validation for ESAT6-based bacterial enumeration. (A) To enumerate the bacterial burden, a standard curve was generated by adding known amounts of Mtb CFUs to age-matched WT lungs and extracting the RNA for measurement of ESAT6 gene expression alongside that of experimental samples as described above and normalized to lung tissue without Mtb. The log2 values of the standard curve were plotted against their respective burden to generate the logarithmic curve. The logarithmic equation of the curve was used to extrapolate the gene expression value of the experimental samples to determine the Mtb burden in CFUs. (B, C) Control and BCG-vaccinated WT and KO mice were challenged with hygromycin-resistant Mtb that constitutively express mCherry providing dual traits that differentiate from BCG. Following 8 weeks of challenge, the left lung was committed to RNA isolation and measurement of ESAT6 expression. The right lung of the same mouse was homogenized for serial dilution and plating on media supplemented with hygromycin. Correlation plots for separate experiments performed at separate times in which symbols correspond to individual mice are shown. [file Image_2.tif]

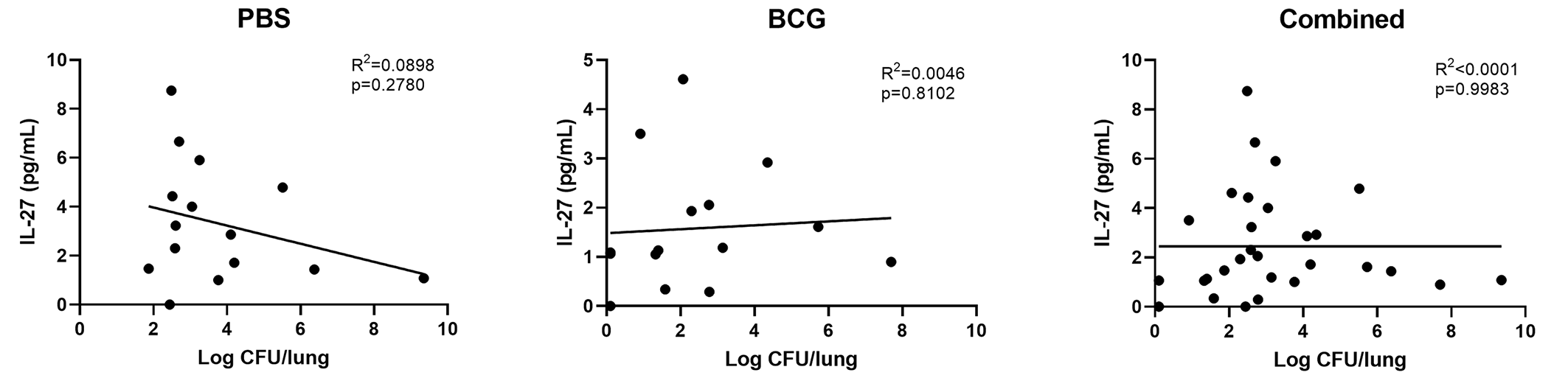

Supplement: Supplementary Figure 3 — IL-27 levels in the periphery do not correlate with control of Mtb in the lungs of mice vaccinated as neonates. Correlation plots of IL-27 serum levels and log10 burdens of Mtb in the lung. Individual symbols correspond to individual mice. The R2 and p values are indicated for the unvaccinated and Mtb challenged mice (A), the BCG-vaccinated and challenged mice (B), or the combined groups (C) respectively. [file Image_3.tif]
